# Oxidative stress and apoptosis after acute respiratory hypoxia and reoxygenation in rat brain

**DOI:** 10.1016/j.redox.2017.02.014

**Published:** 2017-02-24

**Authors:** Debora Coimbra-Costa, Norma Alva, Mónica Duran, Teresa Carbonell, Ramón Rama

**Affiliations:** Department of Cell Biology, Physiology and Immunology, University of Barcelona, Avda Diagonal, 643, 08028 Barcelona, Spain

**Keywords:** Antioxidants, Apoptosis, Normobaric hypoxia, Oxidative stress, Reoxygenation

## Abstract

Acute hypoxia increases the formation of reactive oxygen species (ROS) in the brain. However, the effect of reoxygenation, unavoidable to achieve full recovery of the hypoxic organ, has not been clearly established. The aim of the present study was to evaluate the effects of exposition to acute severe respiratory hypoxia followed by reoxygenation on the evolution of oxidative stress and apoptosis in the brain. We investigated the effect of *in vivo* acute severe normobaric hypoxia (rats exposed to 7% O_2_ for 6 h) and reoxygenation in normoxia (21% O_2_ for 24 h or 48 h) on oxidative stress markers, the antioxidant system and apoptosis in the brain. After respiratory hypoxia we found increased levels of HIF-1α expression, lipid peroxidation, protein oxidation and nitric oxide in brain extracts. Antioxidant defence systems such as superoxide dismutase (SOD), reduced glutathione (GSH) and glutathione peroxidase (GPx) and the reduced/oxidized glutathione (GSH/GSSG) ratio were significantly decreased in the brain. After 24 h of reoxygenation, oxidative stress parameters and the anti-oxidant system returned to control values. Regarding the apoptosis parameters, acute hypoxia increased cytochrome c, AIF and caspase 3 activity in the brain. The apoptotic effect is greatest after 24 h of reoxygenation. Immunohistochemistry suggests that CA3 and dentate gyrus in the hippocampus seem more susceptible to hypoxia than the cortex. Severe acute hypoxia increases oxidative damage, which in turn could activate apoptotic mechanisms. Our work is the first to demonstrate that after 24 h of reoxygenation oxidative stress is attenuated, while apoptosis is maintained mainly in the hippocampus, which may, in fact, be the cause of impaired brain function.

## 1 Introduction

There is abundant literature demonstrating that hypoxia induces increased production of reactive oxygen species (ROS) in brain [1], [2], [3], [4], [5]. ROS are highly reactive and capable of the oxidation of lipids, proteins and DNA [1], [6] leading to structural and functional cellular changes that may cause oxidative injury, apoptosis and necrosis in neurons [7]. In physiological conditions, there is a balance between the formation of ROS and their elimination by the antioxidant system, mainly by superoxide dismutase (SOD), glutathione peroxidase and catalase [8]. Hypoxia modifies the activity of the cytochrome chain responsible for mitochondrial oxidative phosphorylation, resulting in a decrease in ATP synthesis and increased ROS [9] at the same time as a decrease in the activity of the cellular antioxidant system [5], [10], which may lead to oxidative stress. The brain is particularly vulnerable to the effects of ROS for several reasons: (a) it has a low catalase and glutathione peroxidase activity [11], (b) it is rich in lipids with unsaturated fatty acids that can react with ROS and generate peroxyl radicals that oxidize the lipid membrane [12], (c) it has a high metabolic activity and high oxygen consumption [13].

Oxidative stress is a powerful initiator of apoptosis, which also contributes to hypoxia neuronal cell death [14]. Following cerebral hypoxia, the mitochondria release apoptotic proteins such as cytochrome c and apoptosis inducible factor (AIF) into the cytosol [15]. The release of cytochrome c (cyt *c*) and other pro-apoptotic proteins can trigger caspase activation and apoptosis. Once released into the cytosol from the mitochondrial intermembrane space, cyt *c* binds with apoptotic protein-activating factor-1 (Apaf-1) and procaspase-9 to form an “apoptosome”, which actives caspase-9 and subsequently caspase-3. Activated caspase-3 disrupts a wide range of homeostatic, reparative and cytoskeletal proteins and leads to neuron cell death [16]. Upregulation and activation of caspase-3 have been found to precede neuron death in cerebral ischemia [15].

Mammalian cells can adapt to hypoxia. The cellular response to hypoxia is regulated by the hypoxia inducible factor (HIF) [17]. HIF-1 is a heterodimeric transcription factor consisting of an oxygen-regulated HIF-1α subunit and a constitutively expressed subunit HIF-1ß, and functions as a master regulator of oxygen homeostasis in the cell [18]. Although it is degraded under normoxic conditions, in hypoxia its HIF-1α subunit is stabilized and HIF-1 activity rapidly increases [19]. This property makes HIF-1α an excellent marker of tissue hypoxia. Recently, there have been several lines of evidence suggesting that the ROS produced in the mitochondria are responsible for stabilizing HIF-1α during hypoxia [20].

The reoxygenation process after hypoxic insult has been considered as critical for the survival of neurons after hypoxia or ischemia [21], [22]. Although the interchangeable use of the terms hypoxia and ischemia is relatively common [23], [24], they refer to different processes, with different origins and pathological consequences [25]. Hypoxia refers to oxygen deficit in the blood that leads to an oxygen supply that is below tissue requirements. This reduction in oxygen partial pressure induces an increase in cerebral blood flow [26]. In contrast, in brain ischemia, the cerebral blood flow is severely reduced. In hypoxia, though not in ischemia, the cerebral blood flow maintains the supply of glucose, among other substances, and continues to remove metabolic products. Experimental and clinical studies of the effects of reperfusion after brain ischemia have reported dramatic negative effects due to a large increase in ROS levels [27], [28], [29], [30]. The results of this excess of ROS during reperfusion may include the inactivation of detoxification systems, consumption of antioxidants and failure to adequately replenish antioxidants in ischemic brain tissue [31]. Even so, reoxygenation is necessary and furthermore oxygen should be restored within a few hours of the onset of the clinical symptoms of stroke to ensure that as much of the penumbra is rescued [32]. While there have been numerous experimental studies of the effects of reperfusion on brain tissue subjected to ischemia [22], [24], [29], research on hypoxia and subsequent reoxygenation is scarce.

The aim of the present study was to evaluate the effects of exposition to acute severe respiratory hypoxia followed by reoxygenation in brain injury. To this end, we analyzed the redox imbalance and the apoptosis activity in the brain of rats subjected to 6 h in a normobaric hypoxic chamber (7% FIO2) followed by 24 h or 48 h of reoxygenation.

## 2 Materials and methods

### 2.1 Animals and the model of hypoxia

Experiments were carried out using adult male albino Sprague-Dawley rats (Harlam Ibérica, Barcelona, Spain), weighing 230–250 g. The animals were maintained at 22±1 °C with light/dark cycles of 12 h each. The animals had free access to food and water. Experimental procedures were conducted in accordance with the European Community Council Directive of 24 November 1986 (86/609/EEC) under the approval of the Ethical Institutional Committee on Animal Care and Research of the University of Barcelona. Every effort was made to reduce the number of animals used and minimize animal suffering during the experiment.

**Fig. 1:**
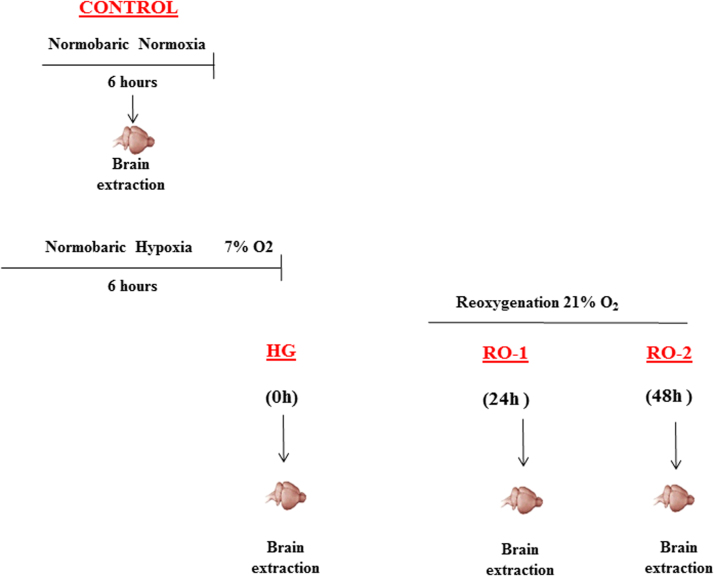
Schematic representation of experimental design. For more details please refer to the material and methods section. HG, hypoxia group; RO-1, 24 h of reoxygenation; RO-2, 48 h of reoxygenation.

For the experimental trial, 54 animals were randomly divided into four groups (Fig. 1): Control group, rats placed in the chamber for 6 h with no exposure to hypoxia; hypoxia group (HG), rats exposed to hypoxia (6 h breathing a gas mixture of 7% O_2_ and 93% N_2_); reoxygenation group (RO1), rats exposed to hypoxia followed by 24 h in normoxia (24 h reoxygenation) and reoxygenation group (RO2), rats exposed to hypoxia followed by 48 h in normoxia (48 h reoxygenation). The fraction of inspired oxygen (FiO_2_) was controlled throughout with an ISO2 World Precision Instruments oxygen sensor (Pyroscience, Bionef, France). During the trial 18.5% of the animals died under hypoxia. The remaining animals were randomly assigned to immunohistochemistry assays or to biochemical and western blot analysis.

For biochemical and western blot determinations, six animals per group were anesthetized with isoflurane inhalation for 1 min and then sacrificed by decapitation. The brain was removed immediately by carefully fracturing the skull from the foramen magnum in the occipital region to the back of the skull. Once extracted, the brains were weighed and immediately washed with ice-cold isotonic saline solution, 0.154 M KCl and placed in liquid nitrogen and stored at −80 °C. The brains were later divided into the two hemispheres and one was used for oxidative stress assays and the other for western blot analyses.

The brain hemispheres used for the different oxidative stress assays described below were first pulverized in a mortar with liquid nitrogen and homogenized.

### 2.2 Measurement of oxidant activity in the brain

#### 2.2.1 Lipid peroxidation

To determine the extent of lipid peroxidation in brain homogenates, thiobarbituric acid-reactive substances (TBARS) were determined using the method described by Mihara and Uchiyama [33] but with minor modifications. Brains were sonicated in 10% (w/v) using a RIPA buffer (Tris 50 mM pH 7.4, 1% Triton X-100, NaCl 150 mM, NaF 5 mM, 0.1% sodium dodecyl sulphate and 1% sodium deoxycholate), to which a protease inhibitor cocktail (Cat. Number P8340 SIGMA-ALDRICH, USA, St. Louis, MO) was added. Homogenates were incubated on ice for 30 min and then centrifuged at 600*g* for 10 min (4 °C). The supernatants were stored at −80 °C until analysis. A malondialdehyde acid (MDA) standard curve was obtained by acid hydrolysis of tetraethoxypropane. The TBA-MDA reaction was carried out by incubation at 95 °C for 10 min. Fluorescence was measured at 515 nm of excitation wavelength (emission at 548 nm). Values for TBARS were expressed in nmol/mg protein.

#### 2.2.2 Advanced oxidation protein products (AOPP)

AOPP is a test that directly measures the amount of oxidized proteins in biological samples. For AOPP determination, tissue was homogenized by sonication in cold buffer containing 50 mM NaH_2_PO_4_ and 1 mM EDTA at pH 7.5. Then, homogenates were centrifuged at 10,000*g* for 10 min (4 °C). Spectrophotometric determination of AOPP levels was performed at 340 nm according to Barsotti's method [34].

#### 2.2.3 Measurement of nitric oxide (NO)

The final and stable end products of NO *in vivo* are nitrates and nitrites, the sum of which (NOx) reflects total NO production [35]. NOx were determined by a colorimetric assay (Cayman Chemical, USA) as described elsewhere [36]. The nitrates in the sample homogenates were enzymatically converted into nitrites by incubation with nitrate reductase, and NADPH and total nitrite (nmol/mg protein) were then monitored with the Griess reaction at 540 nm.

### 2.3 Measurement of antioxidant activity in the brain

#### 2.3.1 Glutathione system

Reduced (GSH) and oxidized (GSSG) glutathione concentrations were measured in brain extracts using the procedure described by Hissin & Hilf [37] with minor modifications [38]. A sample of tissue was homogenized in a cold 1:1 mixture of 0.1 M potassium phosphate, 5 mM EDTA (pH 6.8) and 10% metaphosphoric acid. The homogenate was incubated on ice for 30 min and then centrifuged at 100,000*g* for 30 min. The resulting supernatant was used to determine GSH and GSSG using the fluorescent probe ophthalaldehyde (OPA). Aliquots for GSSG determination were first incubated with N-ethylmaleimide, which complexes GSH, to avoid interference. After a further 15 min of incubation with OPA, fluorescence was determined at 420 nm (excitation 350 nm). The total brain proteins were determined using the Bradford protein assay. For glutathione reductase (GR, EC 1.8.1.7) and glutathione peroxidase (GPx, EC 1.11.1.9) determinations, brains were sonicated in cold buffer containing 50 mM NaH_2_PO_4_ and 1 mM EDTA at pH 7.5. Then, homogenates were centrifuged at 10,000*g* for 10 min (4 °C). Supernatant was used to determine either GR or GPx activity using a Cayman kit (Cayman Chemical, USA Item Number 703202 and 703102, respectively) according to the manufacturer's instructions.

#### 2.3.2 Superoxide dismutase

Brain tissue was homogenized by sonication as described above. Then, homogenates were centrifuged at 600*g* for 10 min (4 °C). The superoxide dismutase (SOD, EC 1.15.1.1.) activity was estimated from the supernatant using an Arbor Kit (Arbor Assay, USA Catalog Number K028-H1).

The activity of all enzymes was expressed as Unit/mg protein.

### 2.4 Western blotting analysis

The brain hemispheres were homogenized in ice-cold lysis buffer containing 50 mM Tris-HCl, pH 7.4, 150 mM NaCl, 1% DOC, 0.1% SDS, 1% Triton X 100, with protease inhibitor cocktail (1:20) (Santa Cruz Biotechnology sc-29130, Santa Cruz, CA, USA). The homogenates were then centrifuged at 12,000*g* for 10 min and the protein concentration measured in the supernatant. Proteins (50 µg) were separated by 8%, 10% and 15% sodium dodecylsulfate-polyacrylamide gel electrophoresis (SDS-PAGE) (Bio-Rad, Hercules, CA, USA) and then transferred to nitrocellulose membranes (Watman® Schleicher & Schuell, Keene, NH, USA). Urea-SDS gels were used to improve the resolution of proteins of low molecular weight. The membranes were blocked in solution containing 5% non-fat milk powder in Tris-buffered saline containing Tween (150 mM NaCl, 20 mM Tris/HCl, pH 7.6% and 0.05% Tween 20) for 1.5 h at room temperature and thoroughly washed with primary antibodies: ß-actin, cytochrome *c* (C20), eNOS (K20), NF-kBp50, HIF-1α (H-206), VEGF, EPO, JNK (D-2), p-JNK, AIF (D20) and iNOS (H-174), all obtained from Santa Cruz Biotechnology (Santa Cruz, CA, USA) and diluted in PBST for 2 h at room temperature. After several washes with PBST, the membranes were incubated for 1.5 h with horseradish peroxidase-conjugated secondary antibodies (Jackson ImmunoResearch, West Grove, PA, USA). Finally, membranes were developed using a kit (Thermoscience), and bands were visualized on x-ray film (Kodak, Rochester, NY, USA). Densitometric analysis was carried out using ImageJ software (NIH, Bethesda, MD, USA).

### 2.5 Immunohistochemistry

Under deep anesthesia using ketamine hydrochloride/xylazine hydrochloride (Ketolar, Parke-Davis, Barcelona, Spain and Rompun, Bayer Healthcare, Kiel, Germany), five rats per group were fixed by intracardiac perfusion with 4% paraformaldehyde in 0.1 M phosphate buffer, pH 7.4. Brains were cryoprotected in a 30% sucrose solution, frozen and sectioned with a Cryostat (Leica Microsystems, Wetzlar, Germany). Immunohistochemistry was performed as described previously [39]. Briefly, histological sections were soaked for 30 min in PBS containing 10% methanol and 3% H_2_O_2_ and subsequently washed in PBS. To suppress nonspecific binding, histological sections were incubated in 10% serum-PBS containing 0.1% Triton X-100, 0.2% glycine, and 0.2% gelatin for 1 h at room temperature. Incubations with the primary antibodies NF-kBp50, AIF (D20) (Santa Cruz Biotechnology, Santa Cruz, CA, USA) and cleaved caspase 3 (Asp175) obtained from Cell Signalling Technology (Denver, CO) were carried out overnight at 4 °C in PBS containing 1% fetal calf serum, 0.1% Triton X-100% and 0.2% gelatin. Antibody binding was detected using the avidin-biotin-peroxidase method (Vectastain ABC kit, Vector Laboratories Inc., Burlingame, CA). The peroxidase complex was visualized by incubating the sections with 0.05% diaminobenzidine and 0.01% H_2_O_2_ in PBS. Sections were mounted, dehydrated, and coverslipped in Eukitt®. Images were obtained with an Olympus BX-61 microscope.

### 2.6 Statistical analysis

Statistical analysis was performed using the Statistical Package for Social Sciences (SPSS), version 13.0. Completion of the Kolmogorov-Smirnov normality test showed normal distribution of mean values of the proteins evaluated. Therefore, we used the parametric one-way ANOVA and post hoc analysis with the Bonferroni test to compare groups. Statistical differences were considered significant when *p*<0.05.

## 3 Results

### 3.1 Brain hypoxia

**Fig. 2:**
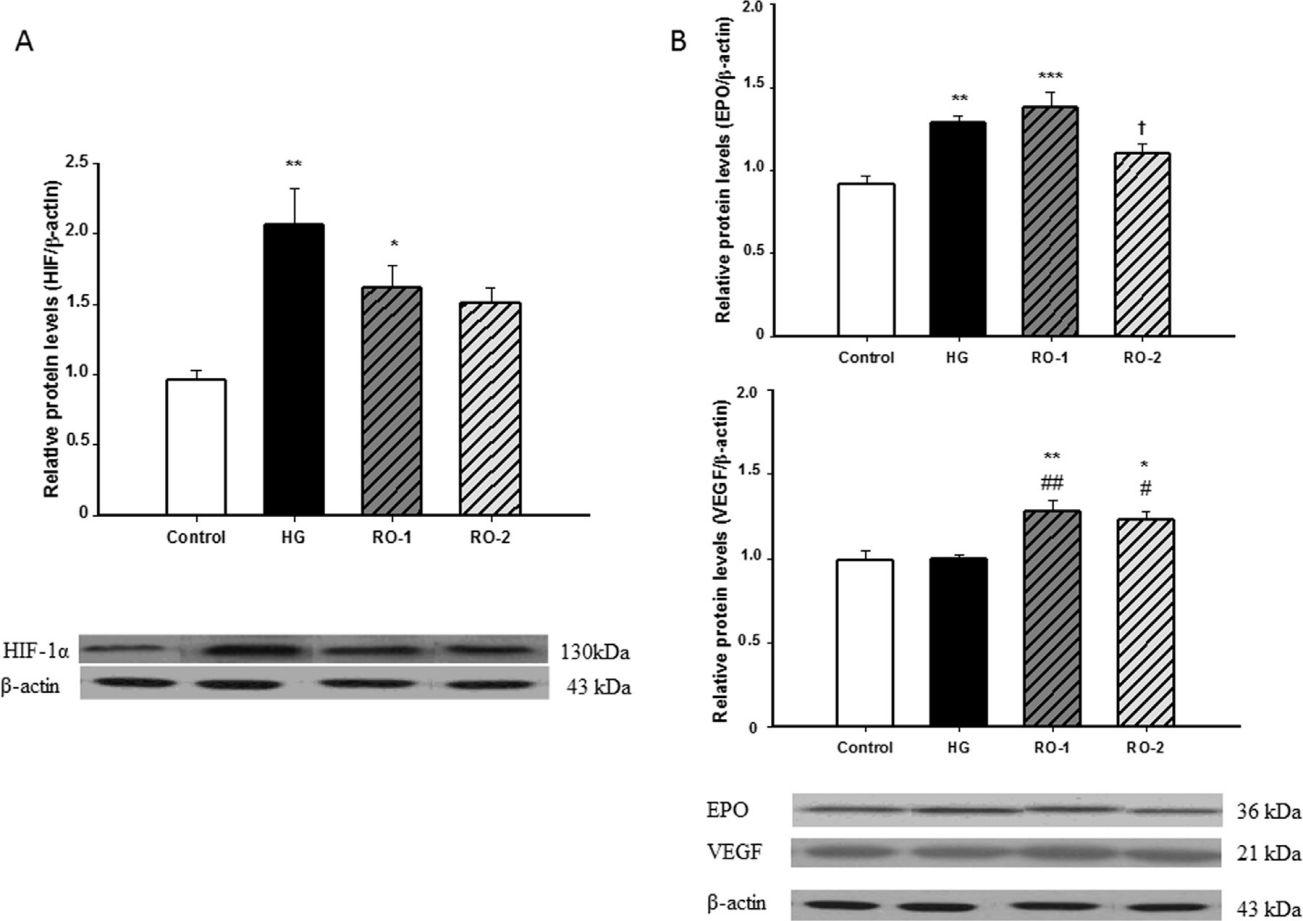
Hypoxia inducible factor (HIF-1α), erythropoietin (EPO) and the angiogenic vascular endothelial growth factor (VEGF). (A) Representative western blots of HIF-1α and β-actin and densitometry ratio of expression in control, hypoxia (HG) and reoxygenation (RO-1, 24 h of reoxygenation and, RO-2, 48 h of reoxygenation) groups. (B) Representative western blots of EPO, VEGF and β-actin and densitometry ratio of expression in control, hypoxia (HG) and reoxygenation (RO-1, 24 h of reoxygenation and, RO-2, 48 h of reoxygenation) groups Results are expressed as relative density units normalized to the control (mean±SEM, n=6). * *p*<0.05 , ** *p*<0.01 and ***p <0.001 represent significant differences *versus* control, # *p*<0.05, ## *p*<0.01 *versus* HG and † *p*<0.05 *versus* RO-1.

As a marker of brain hypoxia, the expression of HIF-1α and the protein levels of two of the main target genes of HIF-1α, the erythropoietin (EPO) and the angiogenic vascular endothelial growth factor (VEGF) were analyzed in control and hypoxic animals. The results showed that exposure to 6 h of severe hypoxia (7% FiO_2_) caused a significant increase in levels of HIF-1α in the brain of hypoxic rats *vs*. brain of normoxic rats that was sustained after 24 h of reoxygenation (Fig. 2A). These results corroborate that this model of 6 h of experimental acute hypoxia is effective at inducing hypoxia in the brain. Moreover, we have measured the protein levels of EPO and VEGF whose functions are to produce adaptations to hypoxia. Our results show different expression levels: while EPO increases with hypoxia and is maintained at RO-1 (mirroring the effects on the expression levels of HIF-1), VEGF has a later response, increasing at RO-1 and remaining high at RO-2 (Fig. 2B).

### 3.2 Oxidative stress in brain after exposure to hypoxia and reoxygenation

**Fig. 3:**
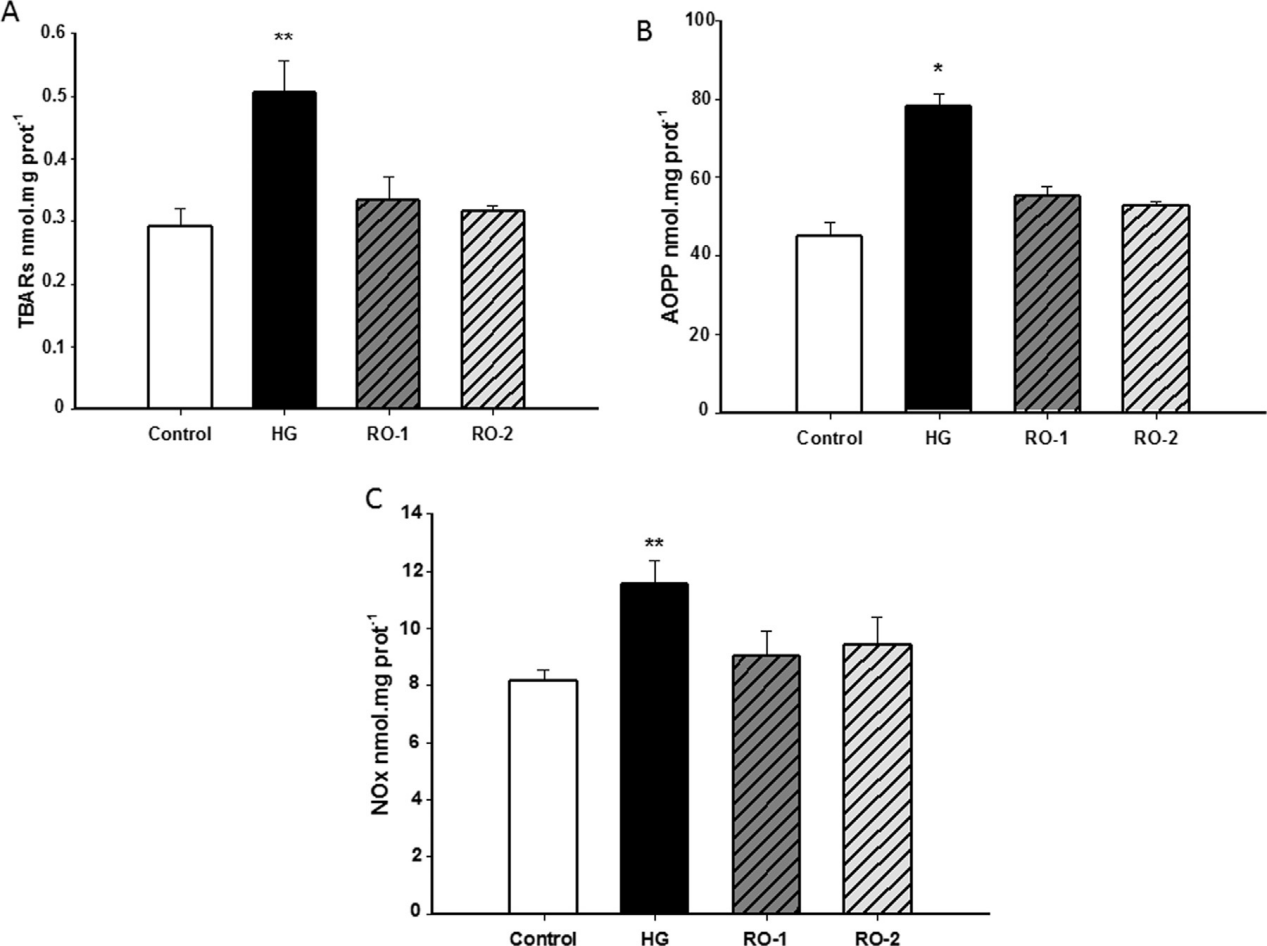
Brain content of oxidative markers. (A) concentration of lipid peroxidation products, TBARs; (B) concentration of protein oxidation products, AOPP; and, (C) nitric oxide derived products, NO_x_ in control, hypoxia (HG) and reoxygenation (RO-1, 24 h of reoxygenation and, RO-2, 48 h of reoxygenation) groups. Results are expressed as mean±SEM (n=6). * *p*<0.05 and ** *p*<0.01 represent significant differences *versus* control.

Hypoxia induced a significant increase (*p*<0.01) in lipid peroxidation products (TBARS) immediately after hypoxia exposure (0.505±0.05 nmol /mg protein in HG group *vs.* 0.292±0.03 nmol/mg protein in control group) (Fig. 3A), while the TBARS values after 24 h (RO-1 group) and 48 h (RO-2 group) of reoxygenation (0.334±0.036 nmol/mg protein and 0.0316±0.009 nmol/mg protein, respectively) did not differ significantly from those in the control group.

A similar response was found in advanced oxidation protein products (AOPP) (Fig. 3B). Immediately after the hypoxia treatment (HG group), the AOPP content in brain was significantly higher (*p<*0.05*)* than that in the control group (78.8±3.13 *vs.* 45.85±4.27 nmol/mg protein in HG and control groups, respectively). Again, no differences were found 24 h (RO-1 group) or 48 h post hypoxia (RO-2 group) (55.4±3.0 and 52.7±1.5 nmol/mg protein, respectively).

The markers of oxidative stress showed that acute exposure to hypoxia caused significant oxidative stress and that after 24 h and 48 h of reoxygenation they had returned to normal values.

Hypoxia induced a significant increase (*p*<0.01) in levels of NOx compared to the control (Fig. 3C): 8.163±0.38 and 11.549±0.83 nmol/mg protein in control groups and HG group, respectively. After 24 h (RO-1 group) and 48 h of reoxygenation (RO-2 group) the NOx content returned to basal values.

**Fig. 4:**
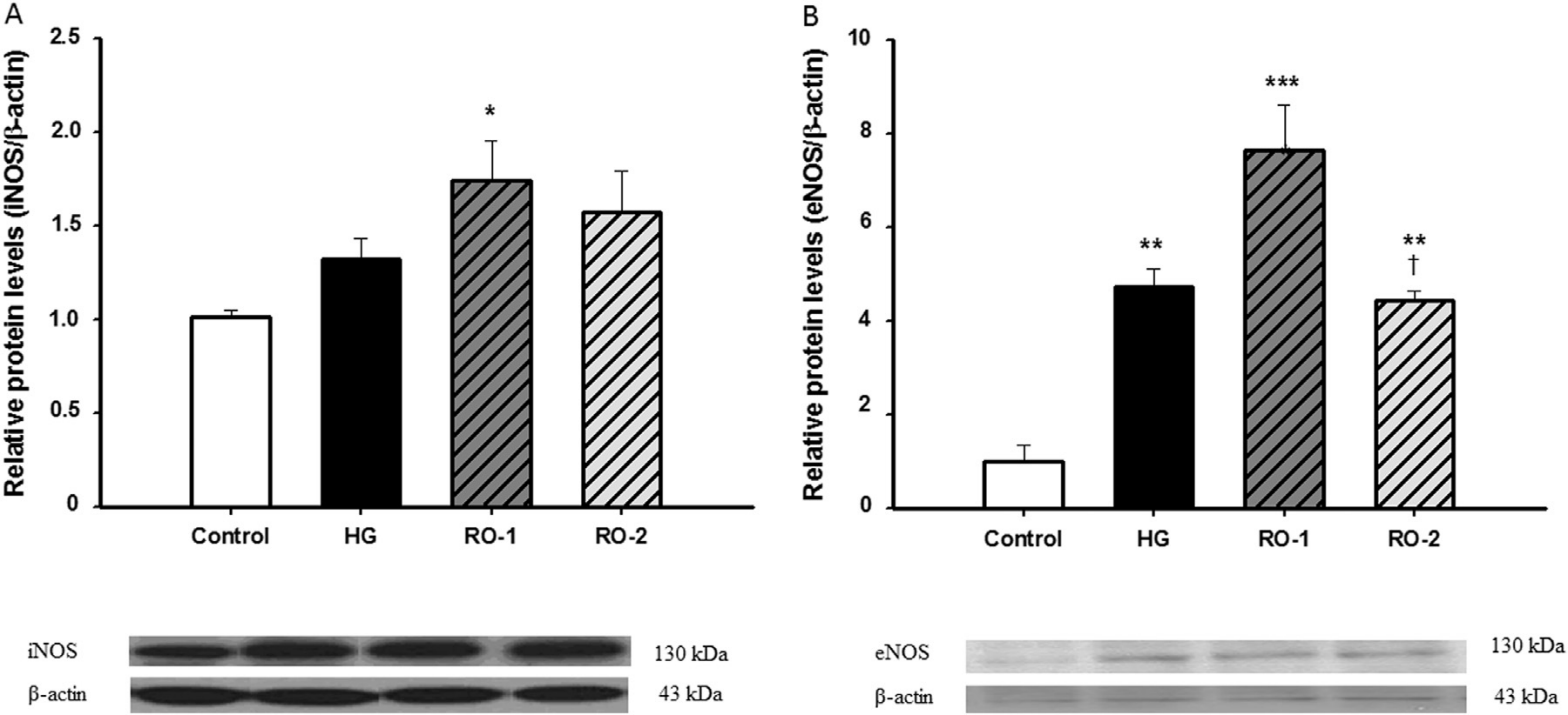
Inducible nitric oxide synthase (iNOS) and endothelial nitric oxide synthase (eNOS). Representative western blots of iNOS (A) and eNOS (B) expression in control, hypoxia (HG) and reoxygenation (RO-1, 24 h of reoxygenation and, RO-2, 48 h of reoxygenation) groups. Results are expressed as relative units as mean±SEM (n=6). * *p*<0.05, ** *p*<0.01 and *** *p*<0.001 represent significant differences *versus* control, and † *p*<0.05 *versus* RO-1.

As for the expression of nitric oxide synthases (NOS), iNOS was not affected by hypoxia treatment but increased expression of iNOS was evident after 24 h of reoxygenation (RO-1 group) relative to the control group (Fig. 4A). In contrast, eNOS expression increased under hypoxia (*p*<0.01, Fig. 4B), as did the NOx level, in the HG group. Expression was still increased at 24 h of reoxygenation (*p*<0.001) but decreased dramatically (*p*<0.05) at 48 h (RO-1 and RO-2, respectively).

### 3.3 Nuclear factor-kappaB (NFkB)

**Fig. 5:**
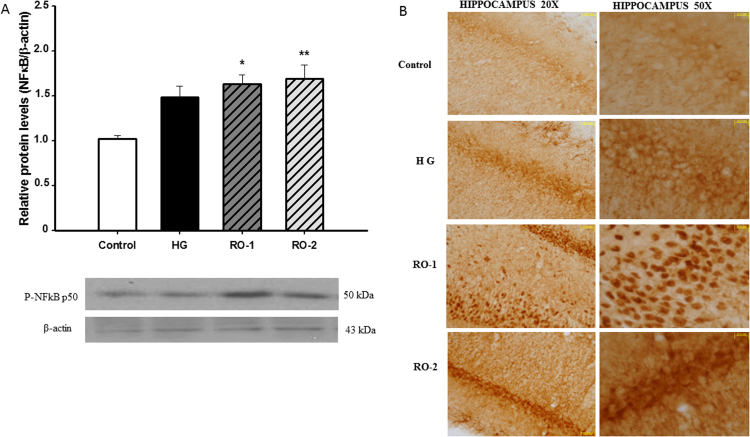
Nuclear factor-kappa B (NFκB). Representative western blots (A) and immunohistochemistry (B) of NFκB in the hippocampus in control, hypoxia (HG) and reoxygenation (RO-1, 24 h of reoxygenation and, RO-2, 48 h of reoxygenation) groups. (n=5). * *p*<0.05 and ** *p*<0.01 represents significant differences *versus* control.

Oxidative stress is a major NFkB-inducing stimulus. We found a significant increase in NFkB expression in whole brain homogenates in RO-1 (*p*<0.05) and RO-2 (*p*<0.01) groups (Fig. 5A). Immunohistochemistry showns NFkB expression in the hippocampus sections of the control group: this expression increased during hypoxia and increased further during reoxygenation (Fig. 5B).

### 3.4 Antioxidant activity in the brain after exposure to hypoxia and reoxygenation

**Fig. 6:**
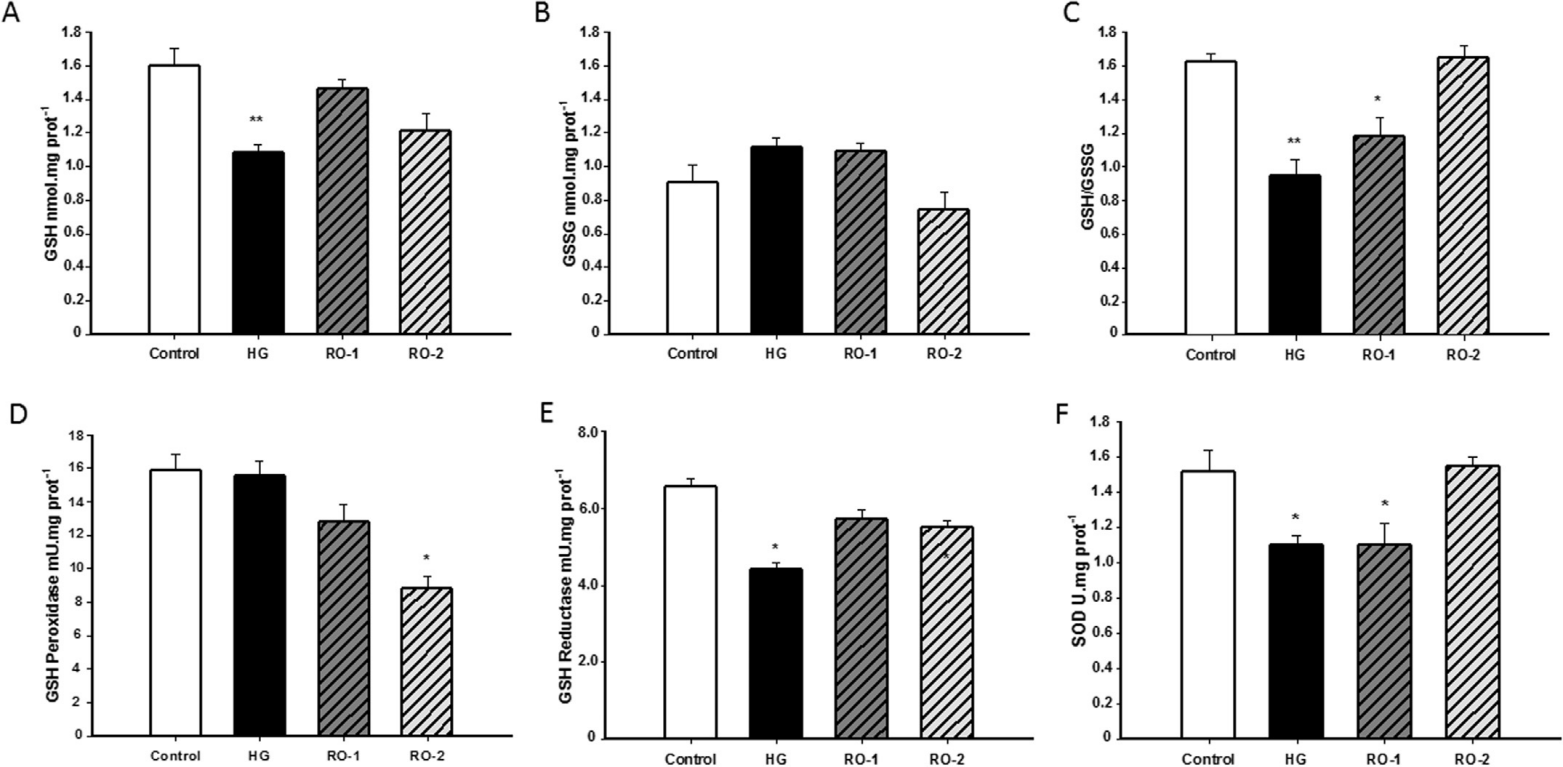
Antioxidants in rat brain: (A) concentration of reduced glutathione GSH, (B) oxidized glutathione*,* GSSG; (C) [GSH]/[GSSG] ratio; (D) GSH-peroxidase activity; (E) GSH-reductase activity and (F) superoxide dismutase activity, SOD expression in control, hypoxia (HG) and reoxygenation (RO-1, 24 h of reoxygenation and, RO-2, 48 h of reoxygenation) groups. Results are expressed as mean±SEM (n=6). * *p*<0.05 and ** *p*<0.01 represent significant differences *versus* control.

Oxidative stress depends on the balance between antioxidant and oxidant elements. Together with increased oxidation of lipids and proteins, the results show that severe acute hypoxia led to a significant decrease in the principal antioxidant molecules in the brain, GSH and SOD. GSH is rapidly oxidized after hypoxia, with a reduction in tissue content of 29% (1.55±0.07 nmol/mg protein in control group *vs.* 1.08±0.04 nmol/mg protein in HG group) (Fig. 6A), and although no changes are observed in GSSG (Fig. 6B) a decrease in the reduced/oxidized GSH/GSSG ratio (1.63±0.05 in control group *vs.* 0.94±0.08 in the HG group) (Fig. 5C) is shown. GSH returned to basal values after 24 h of reoxygenation in RO-1 (94.4%) and the GSH/GSSG ratio also tended to return to basal levels (73.23% and 89.58% of control values at 24 and 48 h of reoxygenation, respectively). No significant changes were observed in glutathione-peroxidase (GPx), which is maintained in hypoxia (Fig. 6D). The decrease in GSH following hypoxia appears to be associated with the decrease in the enzymatic activity of glutathione reductase (GR), the activity of which decreased by 34% following hypoxia (6.56±0.2 mU/mg protein in control group *vs.* 4.39±0.2 mU/mg protein in the HG group) and was restored during reoxygenation (equivalent to 87.2% and 84% of control values in RO-1 and RO-2 groups, respectively) (Fig. 6E).

Another important enzyme with antioxidant activity analyzed in this experiment is SOD, which showed a significant decrease after hypoxia and remained at low levels after 24 h of reoxygenation (1.52±0.09 U/mg protein in control *vs.* 1.10±0.05 U/mg protein and 1.08±0.10 U/mg protein in the HG and 24 h of deoxygenation groups, respectively) (Fig. 6F). SOD activity in brain was found to have returned to basal values 48 h post treatment (1.69±0.15 U/mg protein).

### 3.5 Apoptotic response to hypoxia

**Fig. 7:**
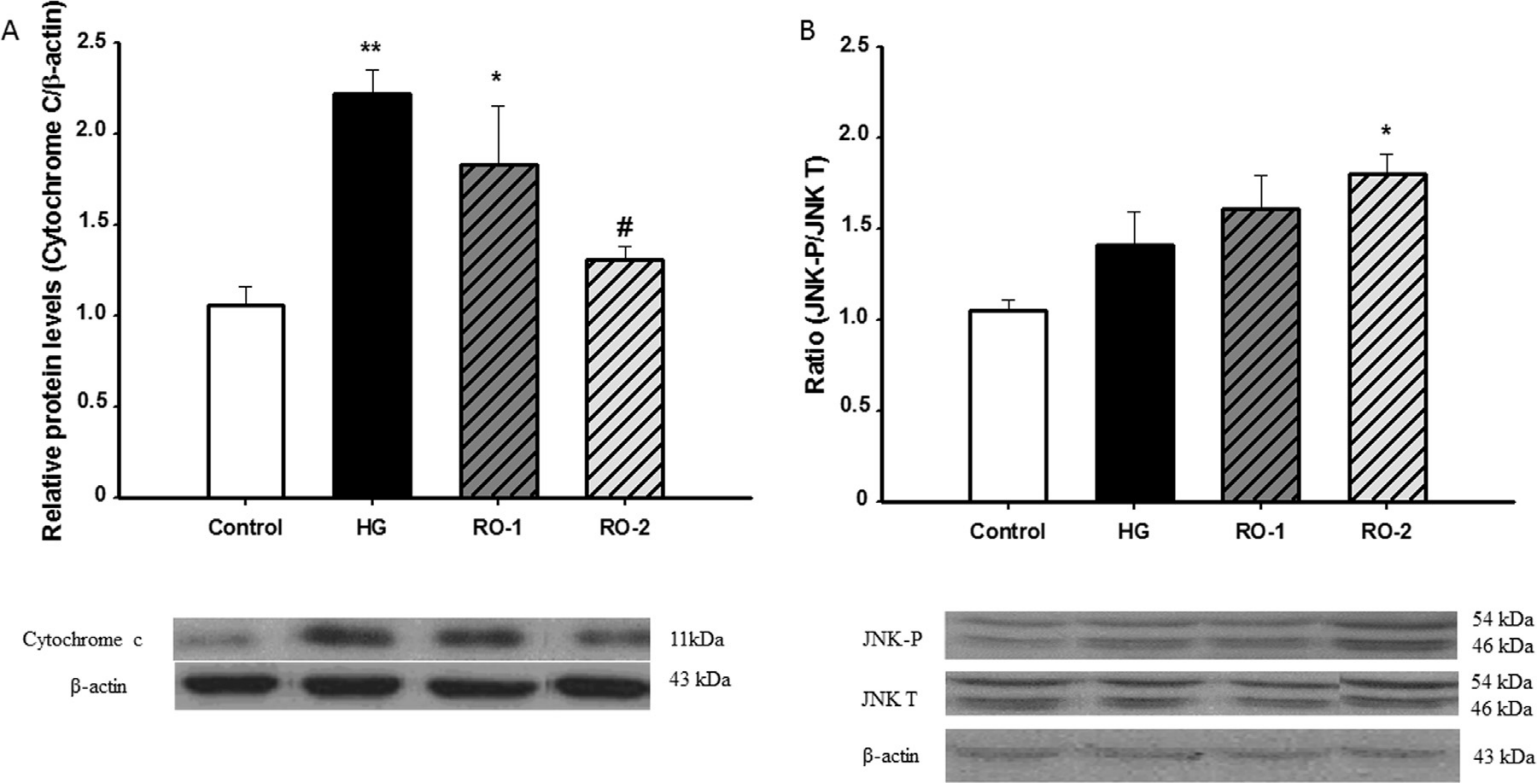
Cytochrome *c* and c-Jun N-terminal Kinases (JNK): (A) representative western blots of cytochrome *c* and β-actin and densitometry ratio in control, hypoxia (HG) and reoxygenation (RO-1, 24 h of reoxygenation and, RO-2, 48 h of reoxygenation) groups. (B) representative western blots of JNK total (JNK-T), phosphorylated JNK (JNK-P) and β-actin and densitometry ratio in control, hypoxia (HG) and reoxygenation (RO-1, 24 h of reoxygenation and, RO-2, 48 h of reoxygenation) groups. Results are expressed as relative density units normalized to the control (mean±SEM, n=6). * *p*<0.05 and ** *p*<0.01 represent significant differences *versus* control and # *p*<0.05 *versus* HG.

Exposure to hypoxia causes a significant increase in cytosolic cytochrome *c* compared with the control group. This increase persisted after 24 h and 48 h of reoxygenation (RO-1 and RO-2) (Fig. 7A). The phosphorylated fraction of c-Jun N-terminal Kinases (JNK) showed a significant increase after 48 h of reoxygenation (RO-2) (Fig. 7B).

**Fig. 8:**
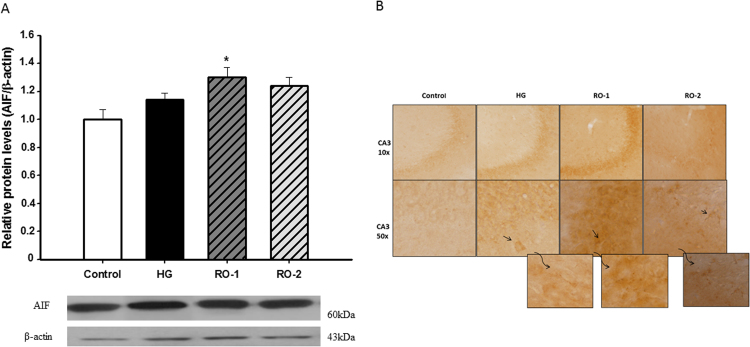
Apoptosis-inducing factor (AIF). (A) Representative western blots of AIF and β-actin and densitometry ratio in control, hypoxia (HG) and reoxygenation (RO-1, 24 h of reoxygenation and, RO-2, 48 h of reoxygenation) groups. Results are expressed as relative density units normalized to the control (mean±SEM, n=6). * *p*<0.05 represents significant differences *versus* control. (B) Representative immunohistochemistry of AIF in the hippocampus and cerebral cortex in control, HG, RO-1 and RO-2 groups (n=5).

Western blotting demonstrated that AIF expression was significantly higher after 24 h of reoxygenation (RO-1) relative to the control group (Fig. 8A). We found that the immunoreactivity of AIF was higher in the CA3 region of the hippocampus and cerebral cortex in brains subjected to 24 h of reoxygenation after hypoxic treatment (RO-1) than in control brains (Fig. 8B).

**Fig. 9:**
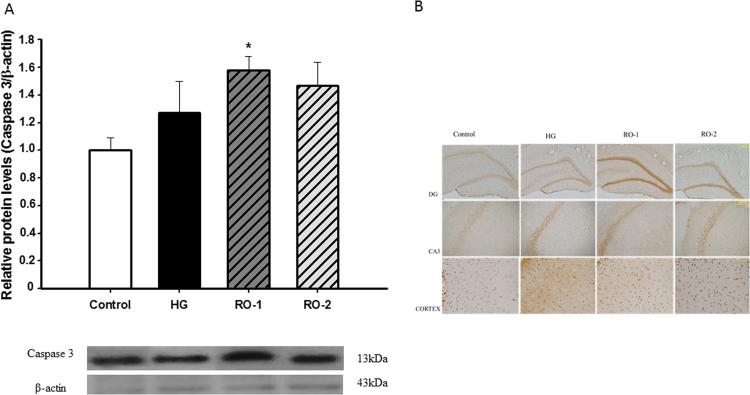
Caspase 3. Representative western blots (A) and immunohistochemistry (B) of caspase 3 in the hippocampus and cortex in control, hypoxia (HG) and reoxygenation (RO-1, 24 h of reoxygenation and, RO-2, 48 h of reoxygenation) groups. (n=5). * *p*<0.05 represents significant differences *versus* control.

The cleaved-caspase-3 protein, an important executor of the apoptotic pathway, was determined by western blot (Fig. 9A) and immunohistochemistry of coronal brain sections (Fig. 9B). The results showed a significant increase in caspase-3 expression in the RO-1 group. Similarly, we observed a slight immunochemistry signal after hypoxia exposure, which was followed by an increase in the RO-1 and RO-2 groups that was more evident in the hippocampal neuron areas CA3 and dentate gyrus (DG) than in the cortex, especially in the RO-1 group.

## 4 Discussion

Neurons are highly susceptible to oxidative stress [40], which is considered the main event induced by hypoxia [2]. Any alteration of pro-oxidant/antioxidant balance can lead to cell damage, among other mechanisms, through an increase in lipid peroxidation, protein oxidation and nitric oxide levels in the cerebral tissues. Unlike what occurs in ischemia, where it has been reported that reperfusion increased oxidative damage [41], in reoxygenation after hypoxia, there is an increase in cerebral blood flow that not only keeps the oxygen supply [42], [43]. but also contributes to the disposal of waste products such as lactate and hydrogen that can cause brain damage [44].

The most important finding of this study is that oxidative damage induced by acute hypoxia is reverted after 24 h of reoxygenation, but in contrast, the apoptotic events, triggered by hypoxia, are maintained during reoxygenation. Such an increase in apoptosis, which is remarkably higher in the hippocampus than in the cortex, may be responsible for the impaired brain functions previously reported in hypoxic brain [45], [46], such as a significant change in spatial reference memory after exposure to hypobaric hypoxia [47].

As a function of the intensity and duration of the hypoxia protocol, oxygen supply to the brain below a critical level can alter oxidative phosphorylation by the mitochondria and drastically reduce cellular ATP production, which results in a rapid decline in cellular ATP to a level insufficient to maintain the activity of ion pumps, in turn leading to rapid and widespread membrane depolarization of neurons and astrocytes [48]. At the same time, mitochondria increase the production of ROS [2], [49], [50].

The present study indicates that TBARS and AOPP (markers of oxidative stress) increase after 6 h of hypoxia exposure. However, reoxygenation causes a progressive decrease in the levels of TBARS and AOPP, indicative of a reduction in oxidative stress. In this model, exposure to acute severe hypoxia causes a loss of antioxidant capacity of brain tissue –*e.g.* decreased superoxide dismutase activity and the reduced form of glutathione– together with increased oxidative damage (*i.e.* increased lipid and protein oxidation). Subsequent reoxygenation causes the recovery of antioxidant activity together with reduced oxidative damage. Antioxidant activity increases compared with its values in hypoxia and returns to normal levels after 48 h in normoxia, while markers of oxidative stress (TBARS, AOPP, and NOx) return to normal levels.

The deleterious effect of ROS in neurons closely interacts with the antioxidant activity thereof. Reduced GSH is a major intracellular antioxidant in the brain, where it is found in higher concentrations than in blood or cerebrospinal fluid [51]. GSH directly scavenges the peroxide of hydrogen resulting from the catalysis of the superoxide radical by SOD. In the present study, hypoxia reduced the amount of GSH in the brain tissue with a consequent reduction of the GSH/GSSG ratio. Under physiological conditions, the activity of glutathione reductase (GR), using NADPH as substrate, recover the GSH levels from GSSG. After 6 h of hypoxia, the GR activity had decreased. The fall in GR activity induced by hypoxia may be the reason why the decline in GSH levels (consumed in the conversion of hydrogen peroxide into water at the onset of hypoxia) was not reversed. This leads to a loss of antioxidant capacity of the glutathione system and increased oxidative stress during hypoxia. After 24 and 48 h of reoxygenation the antioxidant activity and GSH/GSSG ratio reached normal levels respectively. Other authors have also reported similar reductions in GSH activity due to hypoxia, like a drastic 40% decrease after 72 h of hypobaric hypoxia in the cortex of rat brain [47].

Because of the production of superoxide radicals during the oxidative phosphorylation chain in the mitochondria, there is a superoxide dismutase enzyme (SOD) that catalyzes superoxide anion into oxygen and hydrogen peroxide [3] which has an important protective role against induced ROS hypoxia in the cell. The results show that SOD activity was significantly decreased in the hypoxic brain. This reduction was maintained during the first 24 h of reoxygenation and returned to normal values after 48 h of reoxygenation.

Oxidative stress is a major NFkB-inducing stimulus [52]. NFkB is a nuclear transcription factor involved in the regulation of many cellular processes [53], [54]. We found a significant increase in NFkB expression in whole brain homogenates after hypoxia and during reoxygenation. Constitutive expression of low levels of NFkB has been found in brain, and that expression is necessary for cellular activity [55]. In fact, our results show NFkB expression in the hippocampus sections of the control group: this expression increased during hypoxia and increased further during reoxygenation. Crosstalk between ROS and NFkB has been reported, suggesting that it acts as a key negative regulator of oxidant activity induced in response to oxidative stress.

The present study indicates that hypoxia causes an increase in eNOS activity in the brain, as previously observed in hypoxic cardiomyocytes, [56] which remains high during the first 24 h of reoxygenation and returns to normal levels after 48 h of reoxygenation. On the other hand, iNOS expression appears to increase after 24 h of reoxygenation, [57]. The NO^•^ can generate peroxynitrite (ONOO^–^), thus increasing damage due to oxidative stress. An assay using neuron-enriched cultures exposed to ONOO^–^ demonstrated that translocation of apoptosis inducing factor (AIF) into the nucleus was concomitant with impaired mitochondrial respiration and cell death [58]. These events were inhibited when neuron cultures were treated with a peroxynitrite decomposition catalyst, suggesting a central role of ONOO^–^ in mediating the AIF neuron death pathway.

It is well known that excess ROS production, along with its direct action on lipids, proteins and DNA, which leads to damage to cell structures, also causes changes in the mitochondria [59]. Furthermore, the mitochondria can be targeted by their own radicals [60]. Oxidative stress is a powerful initiator of apoptosis, which also contributes to hypoxia neuronal cell death [14]. In the intrinsic pathway, ROS induce mitochondria-dependent apoptosis. This process can be modulated either by AIF or by the release of cytochrome c and the downstream activation of caspases [58], [61].

The results in this study show that hypoxia induces a significant increase in cytochrome c in the cytosol. After 24 h of reoxygenation, the levels of cytochrome c in the cytosol decreased but were even higher than in control animals. After 48 h of reoxygenation cytochrome c levels did not differ significantly from those found in non-hypoxic animals. However, the phosphorylated fraction of JNK showed a significant increase after 48 h of reoxygenation. JNK plays a critical role both in the extrinsic and in the mitochondrial intrinsic apoptotic pathways [62]. Furthermore, it has been reported that JNK is required for the apoptosis of central nervous system neurons [63].

According to the literature, AIF has been proposed to act as a caspase-independent proapoptotic factor induced by ROS released from mitochondria [64]. When AIF translocates from the mitochondria to the nucleus it causes chromatin condensation and DNA fragmentation [65] resulting in neuronal death independent of caspases [66].

A subtle crescent shape of AIF immunoreactivity was found in the CA3 region of the hippocampus and cerebral cortex in the brain of rats subjected to 24 h of reoxygenation after hypoxic treatment. Hypoxia induces an increase in the active form of caspase 3 in the cortex and hippocampus. After 24 h of reoxygenation caspase 3 signal was even greater, particularly in the hippocampus. Caspase 3 activity remained higher after 48 h of reoxygenation, whereas the oxidative stress indicators and antioxidant activity had returned to normal values at this stage. These results suggest that caspase 3 activation could be a delayed effect of oxidative stress in the induction of apoptosis.

The results of acute respiratory hypoxia followed by reoxygenation will eventually include both early oxidative damage and delayed effects that lead to apoptosis. This sequence of events resembles those that occur during ischemic hypoxia and reperfusion [21]. However, during reoxygenation after acute respiratory hypoxia, in contrast what happens during reperfusion after ischemia [67], the oxidant/antioxidant balance recovers and tends to return to normal.

## 5 Conclusions

Our study confirms previous findings that normobaric hypoxia induces lipid and protein oxidation while decreasing antioxidant defense systems in the brain. The main finding of the present work is that, even when oxidative stress precedes the apoptotic cascade, the antioxidant system has been recovered by 24 h post-reoxygenation while, in contrast, the apoptotic cascade is potentiated. In the present model, the sequence of events triggered by acute severe hypoxia may include early oxidative damage, and delayed effects which are conducive to the activation of apoptosis.
